# Gene expression profiling of the Notch-AhR-IL22 axis at homeostasis and in response to tissue injury

**DOI:** 10.1042/BSR20170099

**Published:** 2017-12-22

**Authors:** Marc Weidenbusch, Severin Rodler, Shangqing Song, Simone Romoli, Julian A. Marschner, Franziska Kraft, Alexander Holderied, Santosh Kumar, Shrikant R. Mulay, Mohsen Honarpisheh, Satish Kumar Devarapu, Maciej Lech, Hans-Joachim Anders

**Affiliations:** Nephrologisches Zentrum, Medizinische Klinik und Poliklinik IV, Klinikum der Universität München, Munich, Germany

**Keywords:** crystallopathy, fbrosis, inflammation, ischemia-reperfusion, kidney injury, regeneration

## Abstract

Notch and interleukin-22 (IL-22) signaling are known to regulate tissue homeostasis and respond to injury in humans and mice, and the induction of endogenous aryl hydrocarbon receptor (Ahr) ligands through Notch links the two pathways in a hierarchical fashion. However in adults, the species-, organ- and injury-specific gene expression of the Notch-AhR-IL22 axis components is unknown. We therefore performed gene expression profiling of DLL1, DLL3, DLL4, DLK1, DLK2, JAG1, JAG2, Notch1, Notch2, Notch3, Notch4, ADAM17/TNF-α ADAM metalloprotease converting enzyme (TACE), PSEN1, basigin (BSG)/CD147, RBP-J, HES1, HES5, HEY1, HEYL, AHR, ARNT, ARNT2, CYP1A1, CYP24A1, IL-22, IL22RA1, IL22RA2, IL10RB, and STAT3 under homeostatic conditions in ten mature murine and human organs. Additionally, the expression of these genes was assessed in murine models of acute sterile inflammation and progressive fibrosis. We show that there are organ-specific gene expression profiles of the Notch-AhR-IL22 axis in humans and mice. Although there is an overall interspecies congruency, specific differences between human and murine expression signatures do exist. In murine tissues with AHR/ARNT expression CYP1A1 and IL-22 were correlated with HES5 and HEYL expression, while in human tissues no such correlation was found. Notch and AhR signaling are involved in renal inflammation and fibrosis with specific gene expression changes in each model. Despite the presence of all Notch pathway molecules in the kidney and a model-specific induction of Notch ligands, IL-22 was only up-regulated in acute inflammation, but rapidly down-regulated during regeneration. This implies that for targeting injury responses, e.g. via IL-22, species-specific differences, injury type and time points have to be considered.

## Introduction

Tissue homeostasis has been identified as the key process for survival of multicellular organisms by Claude Bernard in the 19th century [1]. More recently, it has become evident that homeostasis is maintained by a complex interplay between epithelial and immune cells at the tissue level [2]. Tissue injury disrupts homeostasis and induces responses that intend to eliminate the injurious trigger and try to reinstall tissue homeostasis, i.e. inflammation, atrophy, and fibrosis [3,4]. Notch signaling is known to regulate development and homeostasis in diverse organs such as kidney [5], lung [6], heart [7] as well as the intestine [8], and has a central role also in immune homeostasis [9,10]. While therefore the importance of the Notch pathway as a potential therapeutical target is established [11], comparative data for human and mouse organ-specific expression of Notch signalling are lacking, hampering the transferability of murine data to patient-care settings. Albeit some interventions targetting the Notch pathway have been successfully applied in various mouse models of non-renal disease [12–15], the role of Notch signaling in murine renal disease models needs to be further elucidated. Interleukin-22 (IL-22) is a proregenerative cytokine of the *IL-20* gene family [16]) particularly known for immuno-epithelial signaling in regeneration [17,18]. Interestingly, a link between Notch and IL-22 has been shown, as Notch signaling drives IL-22 production via induction of aryl hydrocarbon receptor (Ahr) ligands [19] and IL-22 production *in vivo* is regulated by Notch signaling [20].

Both the Notch [21] and the Ahr/IL-22 [22] pathway comprise *cis*- and *trans*-signaling mediators, respectively. The in-*cis* elements, i.e. Notch ligands are members of the δ and the Jagged gene families [23]. DLL and JAG proteins bind to the in-*trans* expressed Notch receptors Notch1–4 [24], a process that can be inhibited by the endogenous inhibitors DLK1 and DLK2 [25,26]. After ligand–receptor interaction, the matrix metalloproteinase ADAM17 (also known as ‘TNF-α ADAM metalloprotease converting enzyme’ (TACE)) [27] and the aspartate protease PSEN1 [28] cleave the Notch receptor to release its intracellular domain. Also on this level, Notch signaling can be negatively regulated, namely by basigin (BSG, also known as CD147) [29]. After cleavage, the intracellular Notch receptor domain binds to the transcription factor RBP-J [30] to regulate the gene expression of Notch target genes, e.g. HES [31] and HEY [32] proteins. Similarly, the in-*cis* Ahr/IL-22 signaling depends on ligand-induced heterodimerization of AHR with either ARNT [33] or ARNT2 [34]. AHR ligands include exogenous molecules such as 2,3,7,8-tetrachlorodibenzo-*p*-dioxin (TCDD), but also endogenous tryptophan-derivatives and bilirubin. Upon heterodimerization, AHR/ARNT binds to the so-called xenobiotic response elements (XRE) in the promoters of the Ahr target genes *CYP1A1* [35], *CYP24A1* [36], and *I-L22* [37], amongst others. IL-22 is then secreted and binds to the in-*trans* expressed IL22 receptor, which comprises the specific IL22RA1 chain and the common cytokine receptor IL10R2 chain [38]. A soluble decoy receptor termed as IL22RA2 (also known as IL-22-binding protein) acts as a negative regulator at this step of the signaling pathway [39]. Finally, IL-22 effects the in-*trans* target cell that are mediated mainly by STAT3, which after phosphorylation acts as a transcription factor for Ahr/IL-22 signaling target genes [40].

Because of the importance of Notch and Ahr-dependent IL-22 signaling in tissue regeneration upon injury, we speculated on a consistent induction of both pathways upon injury in human and murine tissues.

## Results

### Notch-AhR-IL22 pathway expression in healthy human tissues

**Figure 1:**
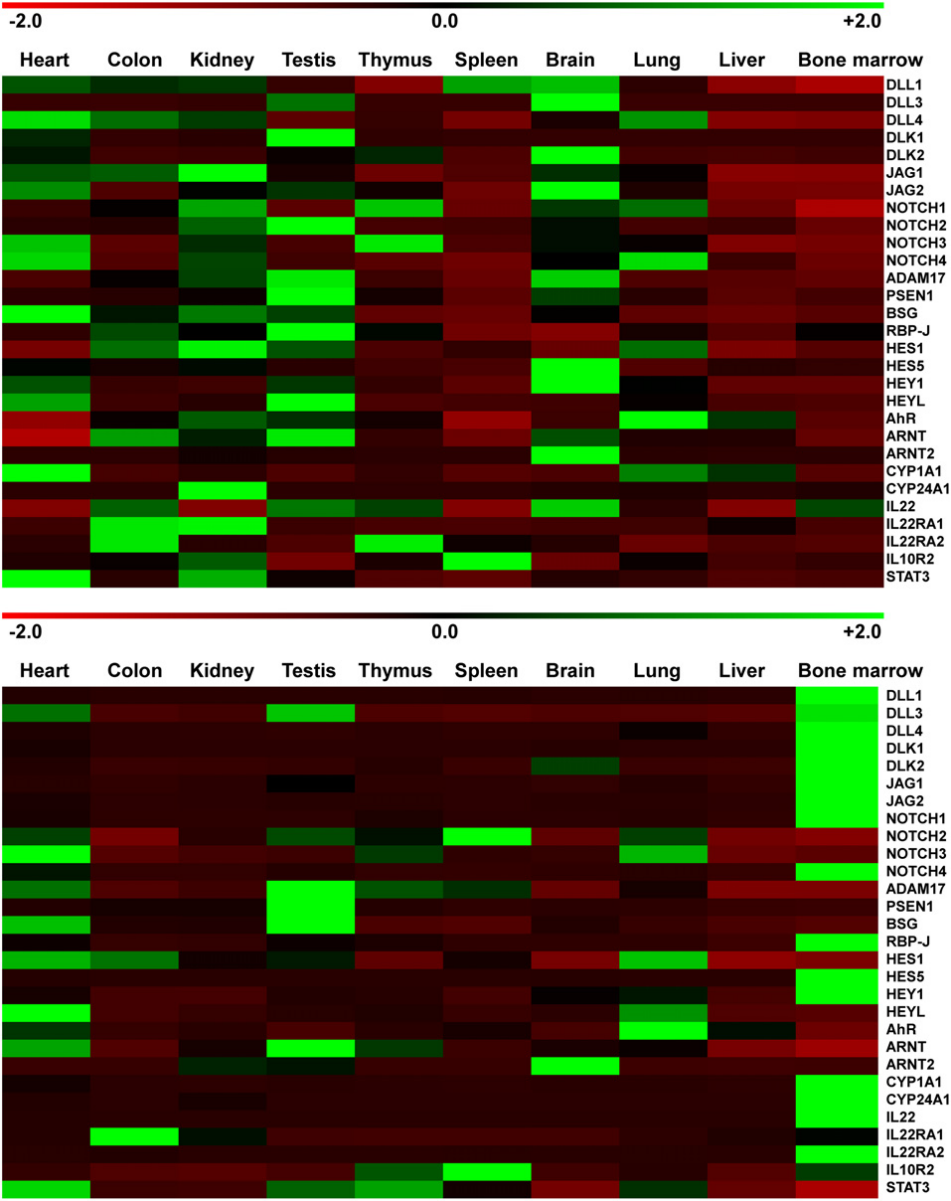
Notch-AhR-IL22 pathway expression in homeostatic human and murine tissues (**A**) Pools of healthy human tissue cDNAs derived from human total RNAs were purified as described in ‘Experimental’ section. Quantitative real-time PCR analysis was performed and mRNA expression levels of all the organs were normalized to *18S* mRNA expression levels and subsequently z-transformed. The table displays red to green shades for higher and lower relative mRNA expression levels, respectively. (**B**) cDNAs derived from five adult 12 weeks old C57BL/6 mice in the same manner are displayed as described in (A).

The relative expression of Notch-AhR-IL22 pathway molecules in ten human organs is shown in Figure 1A. Amongst all the genes, *JAG1, NOTCH2, CD147*, and *STAT3* were most abundantly expressed in all the tissues. Gene expression of pathway molecules was detected in all the tissues, even though expression of HES5 was low in lung and spleen, and DLK1 was low in colon and bone marrow. While myocardial, testicular, and renal tissues showed strongest expression of Notch pathway molecules, the Ahr pathway components showed highest expression in the brain, heart, and kidney. While most organs had high expression of ARNT, which is known to dimerize with Ahr for nuclear signaling, the brain expressed very little ARNT, but had highest expression of ARNT2 amongst all tissues, potentially indicating different Ahr signaling in the brain compared with other tissues.

### Notch-AhR-IL22 pathway expression in healthy murine tissues

In the next step, we characterized expression of the same genes in the murine counterparts of the aforementioned organs. The relative expression of Notch-AhR-IL22 pathway molecules in ten murine organs is shown in Figure 1B. Amongst all the genes, NOTCH1, NOTCH4, CD147, and CYP1A1 were most abundantly expressed in all tissues. Again, gene expression of all pathway molecules was detected in all tissues, even though expression of HES5 and IL22RA2 were low in liver and kidney and DLK1 was low in liver and lung. While murine myocardial, testicular, and hematopoietic tissues showed a stronger expression of Notch pathway molecules, the Ahr pathway components were highly expressed in the heart, testis, and lung. Expression patterns of ARNT and ARNT2 in the mouse closely mirrored the human situation with preferential ARNT expression in all tissues with the exception of the central nervous system, were ARNT2 was more abundantly expressed.

**Figure 2:**
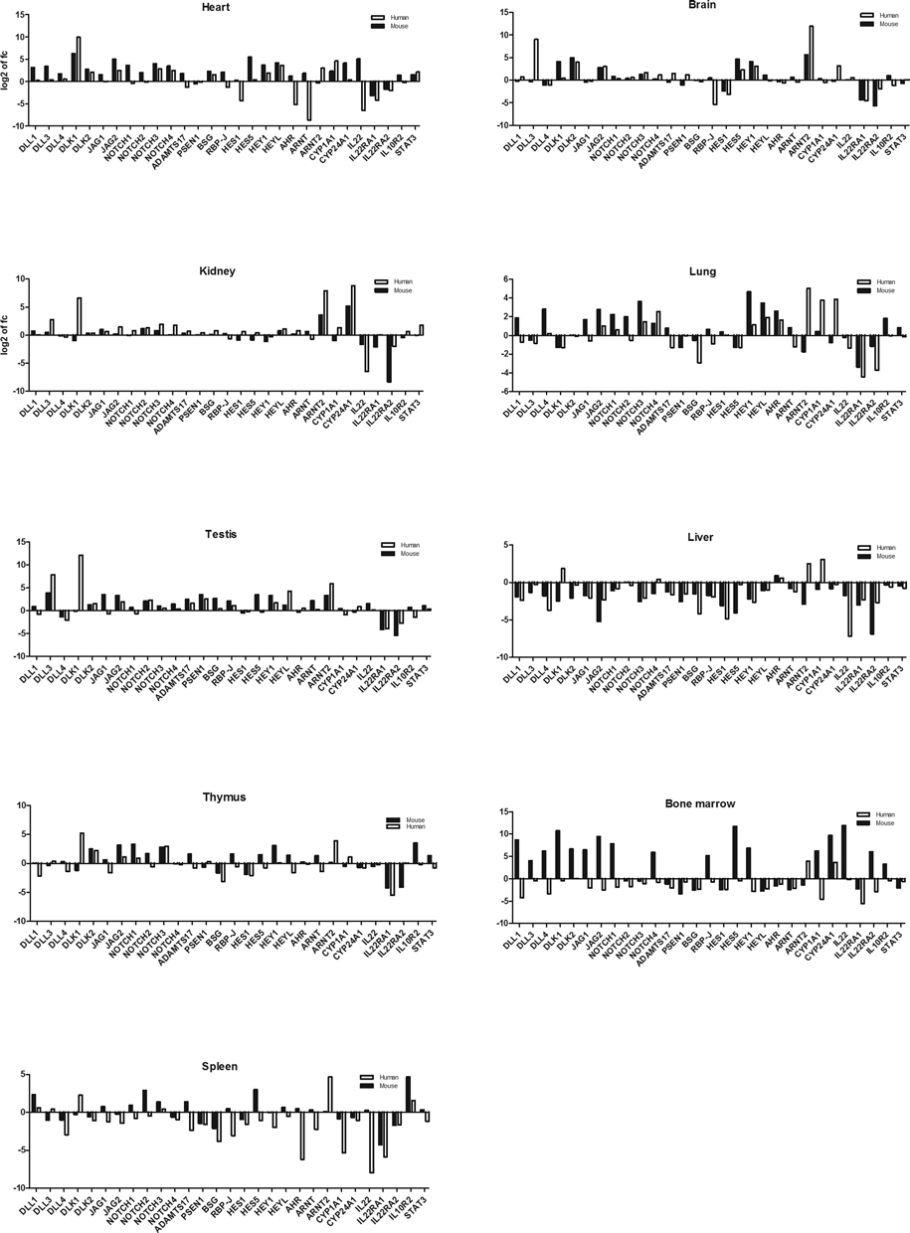
Relative interspecies gene expression of Notch-AhR-IL22 pathway Normalized gene expression to colon. Differential interspecies gene expression was most profound in bone marrow, lung, liver, and thymus.

When we correlated the respective gene expression in humans and mice, we found a significant correlation of expression for all organs with the exception of bone marrow (Table 1). To better compare the relative gene expression between the two species, we sought to normalize gene expression to an intraspecies control and compare normalized expression values. Because of high correlation of absolute gene expression and the very well established concomitant role of both Notch and Ahr signaling in the human and murine gastrointestinal tract, colonic gene expression was selected for normalization. The relative interspecies gene expression is shown in Figure 2. Differential interspecies gene expression was most profound in bone marrow, lung, liver, and thymus, also reflected by non-significant interspecies expression correlations in these tissues after normalization (Table 2). In conclusion, tissue specific gene expression profiles for the Notch-Ahr-IL22 pathway could be detected in murine and human tissues; while these expression profiles correlate well for the colon, kidney, heart, and brain, other organs showed marked interspecies differences.

**Table 1:** Correlation of Respective Gene Expression in Humans and Mice

Organ	R²	p value
Heart	0.9733	< 0,0001
Colon	0.9601	< 0,0001
Kidney	0.9544	< 0,0001
Testis	0.9077	< 0,0001
Thymus	0.7206	< 0,0001
Spleen	0.7154	< 0,0001
Brain	0.5484	< 0,0001
Lung	0.3672	< 0,001
Liver	0.2333	< 0,01
Bone marrow	0.02266	ns

**Table 2:** Correlation of Respective Gene Expression in Humans and Mice

Organ	R²	p value
Heart	0.1715	< 0,05
Colon	n. a.	n. a.
Kidney	0.3616	< 0,001
Testis	0.1721	< 0,05
Thymus	0.1125	ns
Spleen	0.1457	< 0,05
Brain	0.3345	< 0,01
Lung	0.1117	ns
Liver	0.05823	ns
Bone marrow	0.00901	ns

### Notch-AhR-IL22 pathway expression in acute renal inflammation and regeneration

**Figure 3:**
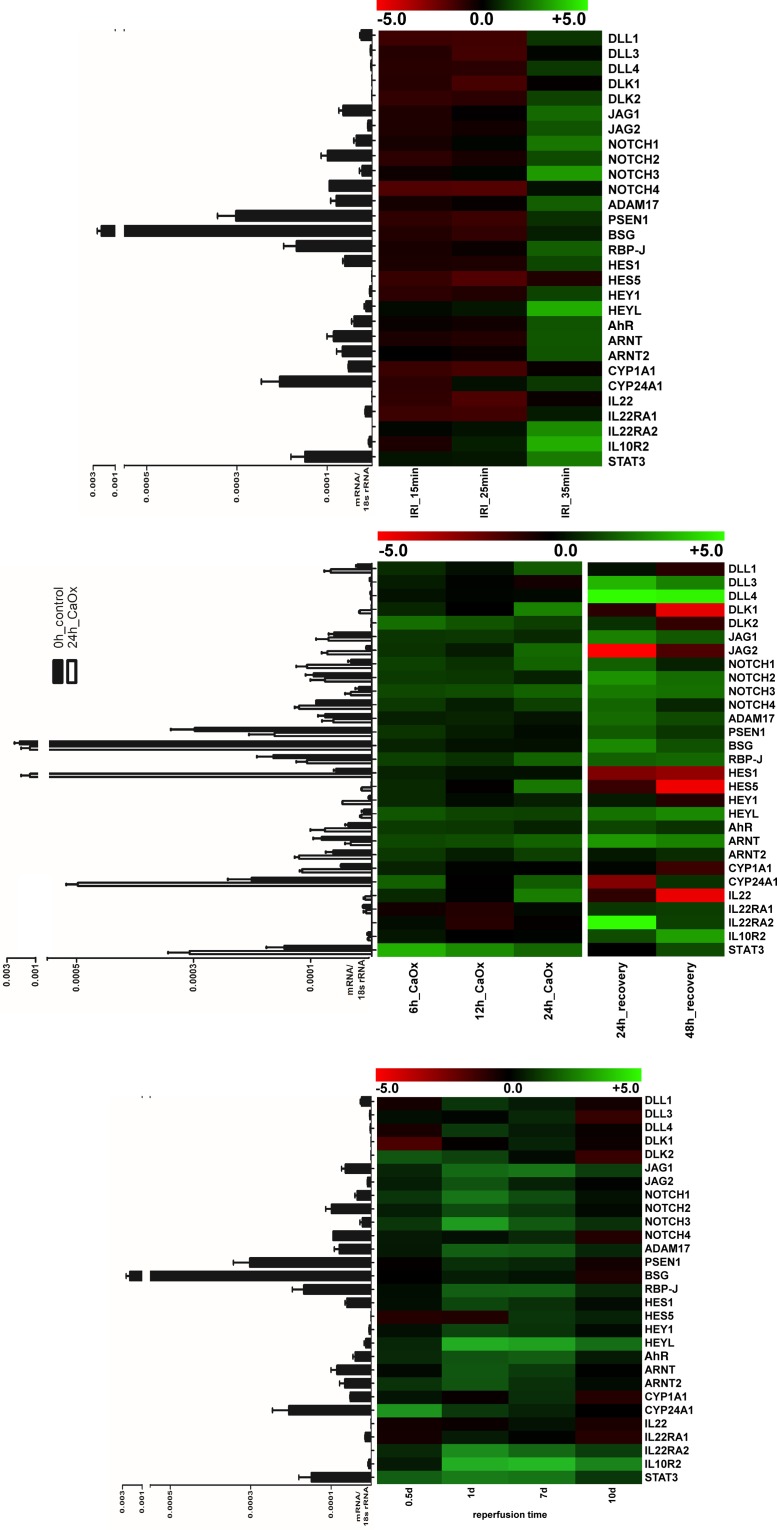
Notch-AhR-IL22 pathway expression in acute renal inflammation and regeneration (**A**) Gene expression for ischemia–reperfusion injury (IRI). A dose-dependent induction of Notch-AhR-IL22 pathway was found in IRI with increasing ischemic time. (**B**) A similar pattern was also seen in AOC after 24 h. The IL-22 expression increased temporally after 24 h of injury and correlated with Hes1 and Hes5. (**C**) Gene expression for IRI regeneration phase. Hes1, Hes5, Hey1, and HeyL all were significantly induced, with HeyL still being up-regulated 10 days after initial injury. Heat maps showing log_2_ fold-changes of the respective sample compared with healthy controls; the table displays red to green shades for higher and lower relative mRNA expression levels, respectively. Bar graphs next to the heat map show absolute levels of respective mRNA expression, normalized to 18S rRNA expression, of healthy murine kidney samples.

**Figure 4:**
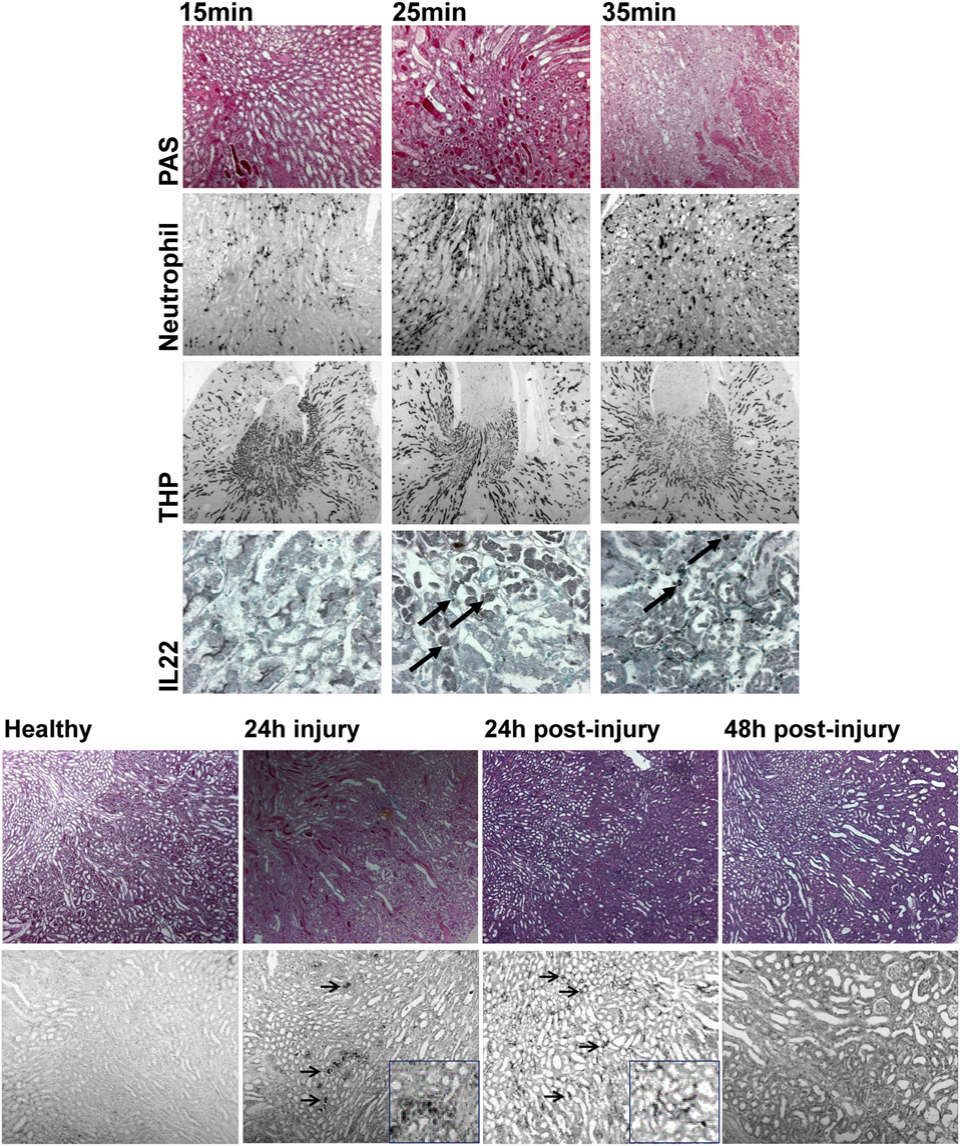
Histological staining of the murine kidneys in acute renal inflammation and regeneration (**A**) rIRI with increasing ischemic time; PAS staining (first row) for evaluation of general injury (e.g. hyaline casts), IHC for evaluation of neutrophil influx (indicating intrarenal inflammation) (second row), anti-THP for intact distal tubular cells (third row), and anti-IL22 for IL-22 positive cell enumeration. In rIRI models, with the ischemic time increasing, hyaline material, neutrophil- and IL-22 positive cell numbers increased while THP staining decreased. Magifications are 100× for THP, 200× for PAS and neutrophil staining, and 400× for IL-22 staining. (**B**) PAS staining in rAOC showing the hyaline material (i.e. injury) peak after 24 h of oxalate injury and a steady decrease post-injury thereafter, along with a similar course for IL-22 positive cells. Magifications are 200× for PAS and IL-22 staining; insets are 400×. Arrows are indicating IL-22 positive cells in the renal interstitium.

After characterizing the baseline expression of the Notch-AhR-IL22 pathway in homeostatic human and murine tissues, we next sought to analyze dynamic changes of the pathway under pathological conditions. As good correlations for intrarenal murine and human gene expression were found and roles for both Notch and Ahr/IL-22 pathways have been established in the kidney, we chose two murine models of acute renal inflammation to study Notch-AhR-IL22 pathway expression. The absolute and relative gene expressions for renal ischemia–reperfusion injury (rIRI) and acute renal oxalate crystallopathy (rAOC) are shown in Figure 3A,B. Interestingly, a dose-dependent induction of the Notch-AhR-IL22 pathway was found in rIRI with increasing ischemic time. Most profound up-regulation was seen 24 h after 35 min of rIRI for JAG1, JAG2, NOTCH1–3, and HEYL as well as for IL22RA2, IL10R2, and STAT3 (Figure 3A). A very similar pattern of gene expression was also seen in rAOC after 24 h (Figure 3B). Importantly, while in rIRI IL-22 was down-regulated, in rAOC IL-22 gene expression peaked after 24 h of injury. This increase was only temporarily, as IL-22 levels decreased again in the regeneration phase. Of note, the IL-22 expression in rAOC correlated with the Notch target genes *Hes1* and *Hes5*, which also showed transient up-regulation followed by prolonged down-regulation during the 24 and 48 h of regeneration after injury. Interestingly, this pattern was not observed in the regeneration phase after rIRI, where Hes1, Hes5, Hey1, and HeyL all were significantly induced, with HeyL still being up-regulated 10 days after initial injury (Figure 3C, for graphic representation of fold-changes after rIRI also see Suppl. Fig. 1). With increasing ischemic time, periodic acid–Schiff’s staining (PAS) at the corticomedullary junction (i.e. the maximum damage zone) showed more general injury (e.g. more hyaline material within renal tubules) as well as more intrarenal inflammation indicated by a higher neutrophil influx. Concomitantly, there were decreased intact distal tubular cells, indicated by less Tamm–Horsfall protein (THP) positive cells. Consistent with increasing inflammation, the number of IL-22 producing cells increased with ischemic time (Figure 4A). In rAOC, hyaline material within tubules in the entire kidney peaked after 24 h of oxalate injury, and then decreased after 48 h post-injury. Again, there was similar pattern for intrarenal IL-22 expression in these samples with IL-22 positive cells peaking at 24 h of oxalate injury and decreasing thereafter (Figure 4B). In conclusion, several members of the Notch-AhR-IL22 pathway are differentially regulated during specific phases of acute kidney injury and regeneration.

### Notch-AhR-IL22 pathway expression in chronic tubular atrophy

**Figure 5:**
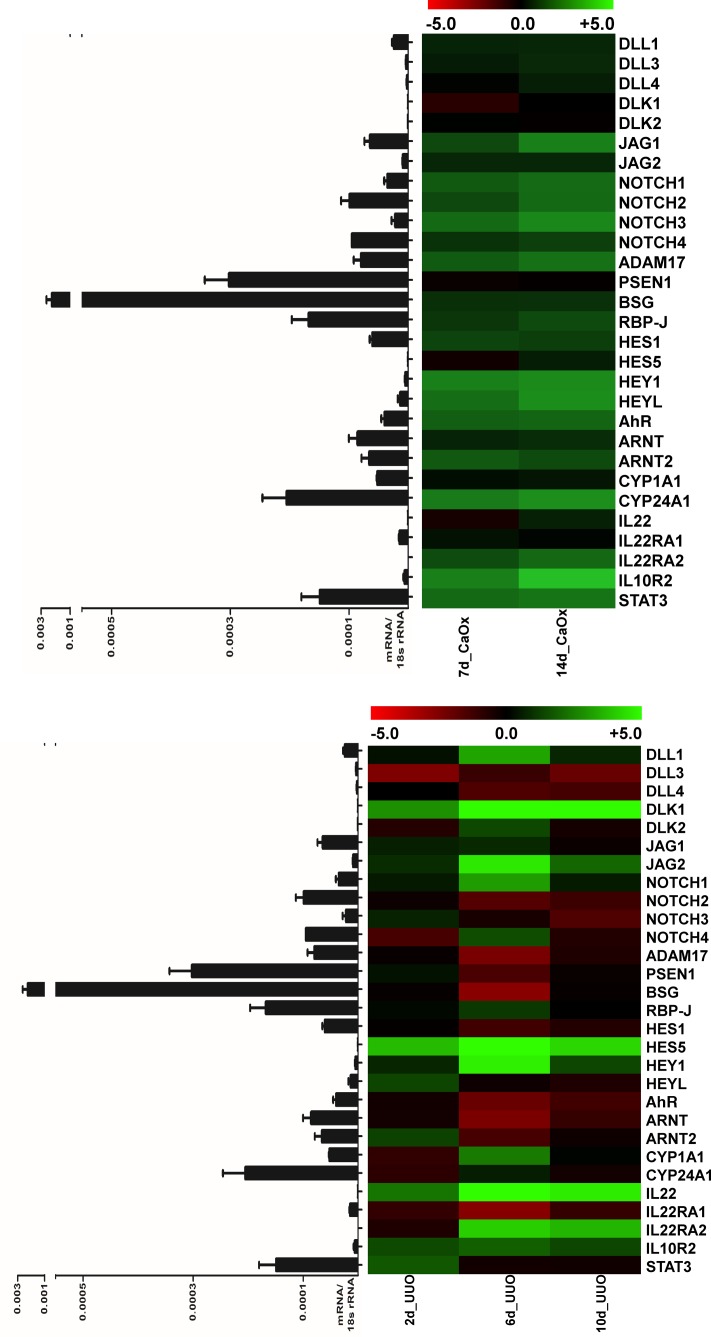
Notch-AhR-IL22 pathway expression in chronic tubular atrophy (**A**) Gene expression pattern in rCOC models. Notch4 was up-regulated after 7 days of rCOC and stayed up-regulated, Notch1 and Notch2 were up-regulated only after 14 days, while Notch3 was not significantly regulated. Hey1 was the most highly induced gene after 7 and 14 days. Both Ahr and Arnt2 were induced, while Arnt was not regulated. (**B**) Gene expression pattern in UUO models. A strong up-regulation of Hes5 and IL-22, was seen at all time points. Heat maps showing log_2_ fold-changes of the respective sample compared with healthy controls; the table displays red to green shades for higher and lower relative mRNA expression levels, respectively. Bar graphs next to the heat map show absolute levels of respective mRNA expression, normalized to 18S rRNA expression, of healthy murine kidney samples.

**Figure 6:**
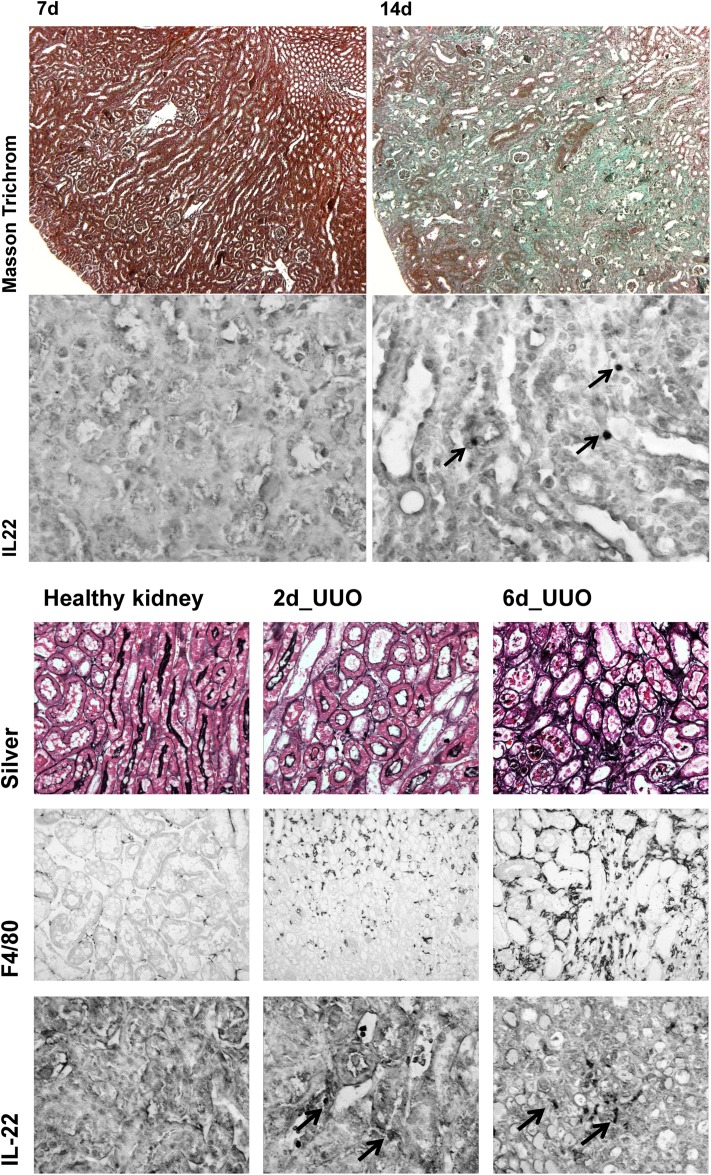
Histological staining in chronic tubular atrophy (**A**) In rCOC models, Masson’s trichrome staining showed more fibrosis (green) in kidney after 14 days of rCOC, meanwhile, IL-22 staining also increased after 14 days of rCOC. Magnifications are 200× for Masson’s trichrome and 400× for IL-22 staining. (**B**) Silver and F4/80 stainings in the UUO model show increasing fibrosis (indicating worsening kidney function) and macrophage influx (indicating persisting inflammation) along with increasing numbers of IL-22-expressing cells. Magnifications are 200× for Silver, F4/80, and IL-22 staining. Arrows are indicating IL-22 positive cells in the renal interstitium.

Acute inflammation is followed by regeneration to restore tissue homeostasis, but if the injurious trigger persists, irreversible damage of tissues occurs. In the kidney, this leads to epithelial atrophy, which is accompanied by interstitial fibrosis caused by maladaptive repair mechanisms. We therefore next analyzed two mouse models of chronic tubular injury and atrophy. While short exposure to oxalate crystals induces reversible rAOC, long-term oxalate overload induces renal chronic oxalate crystallopathy (rCOC) like in primary hyperoxaluria type 1. Another well characterized model of chronic kidney disease (CKD) is unilateral ureteral obstruction (UUO), in which intraluminal hydrostatic pressure induces the tubular atrophy. Gene expression patterns of the Notch-AhR-IL22 pathway in both models are shown in Figure 5A,B. While Notch4 was up-regulated already after 7 days of rCOC and stayed up-regulated thereafter, Notch1 and Notch2 were up-regulated only after 14 days of rCOC, while Notch3 was not significantly regulated. Amongst the most highly induced, both after 7 and 14 days of rCOC was Hey1, with six- and eight-fold up-regulation, respectively. Interestingly, while both Ahr and Arnt2 were induced in rCOC, Arnt was not regulated. The gene expression pattern in UUO was somewhat distinct from the one seen in rCOC. In UUO, a strong up-regulation of Hes5, concomitant with up-regulation of IL-22, was seen at all time points. In conclusion, also in chronic tubular atrophy specific regulation of the Notch-AhR-IL22 pathway was regulated, albeit less pronounced than in the acute injury models. Histological staining in chronic tubular atrophy showed diffused increasing renal fibrosis (extensive green-bluish areas in Masson’s trichrome staining) after 14 days of rCOC, and a moderate increase in IL-22 positive cells after 14 days of rCOC (Figure 6A). Silver and F4/80 stainings in the UUO model show increasing fibrosis (indicating worsening kidney function) and peritubular macrophage influx (indicating persisting inflammation) along with increasing numbers of IL-22 expressing cells (Figure 6B), after ureteral ligation over time.

## Discussion

The Notch and the Ahr/IL-22 signaling pathways are well described and recently, a hierarchical connection of the two pathways has been described [19,20]. Relatively little is known about differential expression the pathway between mice and men. When we compared the expression of 29 members of the Notch-AhR-IL22 pathway in ten murine and human tissues, overall similarities were identified, albeit with some relevant differences. Except for the bone marrow, gene expression relative to 18S rRNA was significantly correlated between murine and human tissues. Out of all the tissues analyzed, expression of the Notch-AhR-IL22 pathway is best characterized both in men and mice within the colon [41,42]. When gene expression was further normalized to the colon, interspecies correlation remained significant only for heart, kidney, testis, spleen, and brain. Amongst these organs, the renal gene expression showed the highest correlation (Pearson’s *r*² =0.36). While most organs had high expression of ARNT, which is known to dimerize with Ahr for nuclear signaling, the brain expressed very little ARNT, but had highest expression of ARNT2 amongst all the tissues, potentially indicating different Ahr signaling in the brain compared with other tissues. This is particularly interesting, as in rCOC, chronic injury strongly induced ARNT2 along with Ahr expression, probably leading to altered downstream signaling of the Ahr pathway. When we analyzed the correlation between Notch and Ahr/IL-22 target gene expression, the Ahr-dependent ligand expression correlates less with Notch target genes in humans than in mice. As the difference is most pronounced in lung and liver, probably exogenous Ahr ligand from human environments not present in relatively enclosed laboratory environments account for this phenomenon.

In the present paper, we not only characterized homeostatic gene expression, but also analyzed expression patterns of the Notch-AhR-IL22 pathway in different models of acute and chronic injury. As Kulkarni et al. [43] and Bielesz et al. [44] have shown roles of Notch and IL-22 in kidney disease, it was not surprising to find several elements of the Notch-AhR-IL22 pathway differentially expressed under pathological conditions. Most notably, an upregulation of both Notch ligands as well as Notch and Ahr target geneswas observed in acute and chronic kidney injury models. When we compared gene expression patterns of the Notch-AhR-IL22 pathway between acute and chronic injury in detail, several differences were found: (i) while in acute models there was a transient up-regulation of JAG1 and JAG2, followed by a down-regulation of these genes during recovery, in chronic injury members of the δ-like family of Notch ligands were persistently induced, (ii) while in acute rIRI AHR was up-regulated along with ARNT, in rCOC it was up-regulated with ARNT2, and (iii) significant IL-22 induction was only seen in rAOC after 24 h of injury concomitant with Hes5 expression.

While for IL-22, we provide histological evidence for consistent mRNA and protein expression, the data provided here mostly relied on mRNA expression as assessed with RT-qPCR. While we can provide compelling evidence for regulation of specific genes of the Notch-AhR-IL22 pathway, obviously these results should be validated on the protein level. Beyond that, based on the design of our study, we cannot prove any causal role for the genes being identified in the different models. As some of our observations are in agreement with already published roles of single genes [44], it would be very interesting to see, whether other yet unvalidated findings of the present study will translate into the discovery of causal roles of the respective genes in each model.

## Experimental

### cDNA preparation from human solid organs

We procured human total RNA Master panel II (Clontech) and selected an array of human solid organs. The used modified guanidinium thiocyanate method [45,46] ensured a pure preparation of DNase-treated RNAs from tissues of healthy male and female humans (Cohort, see Supplementary Table S1) and purity was assessed by UV spectrophotometry. Representative tissue samples or whole organs were used for RNA isolation and selection bias was reduced by pooling samples from different human individuals for most of the organs. cDNA was obtained by reverse transcription. In detail, we created a master mix consisting of Superscript II reverse transcriptase (Invitrogen), hexanucleotides, DTT, linear acrylamide, dNTPs, and 5× Superscript buffers according to established protocols [47]. One microgram of RNA sample per reaction was transcribed into cDNA at 42°C within 90 min. For PCR analysis equal amounts of cDNA were used and 18S rRNA served as a housekeeping gene during the experiments. Since the purchased human samples were single pools, it was not possible to calculate statistics of biological replicates. Video imaging and computer analysis was used to determine the PCR product band and the band intensity was assessed. If the average band intensity for the reference genes varied for more than 20%, the concentration of individual cDNA preparations was then adjusted accordingly.

### cDNA preparation from murine solid organs

Twelve weeks old adult C57BL/6 mice were ordered from Charles River, Sulzfeld, Germany. Mice were housed in groups of five in polypropylene cages under specific pathogen-free conditions with free access to food and water. They were kept under standard conditions in a 12-h light/dark cycle. Under general anesthesia, mice were killed by cervical dislocation and tissues were immediately transferred to RNAlater (Ambion, Carlsbad, CA, U.S.A.). Ten milligrams of tissue mass was used to obtain high quality, DNA-free, RNA with Pure Link RNA Mini Kit (Ambion, Carlsbad, CA, U.S.A.) according to manufacturer’s instructions, to ensure high-quality DNAse solution was applied to each sample and traces of DNAse were eliminated by additional washing steps. NanoDrop 1000 spectrophotometer (PEQLAB Biotechnologie, Erlangen, Germany) was used to assess the concentration and purity of aqueous RNA samples as described [48,49]. In brief, the absorbance ratio 260/280 was calculated and values between 1.95 and 2.05 were regarded as pure RNA. In order to generate cDNA, 1 μg of high-quality RNA of each individual sample was transcribed into cDNA using thermostable RNAse inhibitor during reverse transcription as described above.

### Animal models of acute and chronic tissue inflammation

Groups of 8-week-old C57BL/6 mice (*n*=5) were flank incised under anesthesia and the renal artery of one kidney was clamped for 15, 25, and 35 min. Afterward, kidneys were reperfused for 12, 24 h, 7, and 10 days, a well-established [50] model of ischemia–reperfusion injury (IRI). Heating pads provided a constant body temperature of 37°C throughout the procedure assessed by rectal temperature controls [51]. In order to induce acute CaOx nephropathy, 6- to 8-week-old C57BL/6 mice were injected with a single dose of 100 mg/g sodium oxalate (Santa Cruz Biotechnology, U.S.A.) and were provided with 3% sodium oxalate drinking water as previously described [52]. We modified our established protocol by harvesting the kidneys after 6, 12, and 24 h. For regeneration studies, mice received normal drinking water after 24 h of exposure to CaOx drinking water and kidneys were collected 24 and 48 h later. Chronic CaOx nephropathy and UUO were induced as described [53,54]. All animal experimental procedures were performed according to the German Animal Care and Ethics Legislation and had been approved by Regierung von Oberbayern.

### Quantitative real-time RT-PCR

The mRNA expression levels of the AhR/Notch/IL-22 axis in human and murine solid organs were assessed by quantitative RT-PCR. Ribosomal 18S rRNA was used to normalize mRNA expression of target genes. In order to control sample homogeneity geometric mean (GM), arithmetic mean (AM) minimal value, maximal value, S.D., variance, and coefficient of variance (CV) of the housekeeping gene 18S were calculated (Supplementary Table S2+ S3). According to established protocols [55,56], each PCR reaction (20 μl) comprised gene-specific primers, dNTPs, 10× Taq Polymerase buffer, Taq Polymerase, PCR Optimizer, BSA, SYBR green solution, MgCl_2_, and 0.2 μl of synthesized cDNA. Amplification and detection of cDNA was performed on SYBR Green Dye detection system (SYBR Green I 96 protocol LC480 Roche running program, Roche, Penzberg, Germany). For all quantitative real-time PCR experiments LightCycler 480 (Roche, Mannheim, Germany) was used. Our established protocol [47] comprising initiation phase at 95°C, annealing phase at 60°C, and amplification phase at 72°C was performed for a total of 40 amplification steps. Gene-specific and temperature-optimized primers (Supplementary Tables S4, S5) were purchased from Metabion, Martinsried, Germany after careful *in silico* specificity screen (BLAST). Primer design ensured specificity for cDNA transcripts and covered most CCDS-approved transcripts. Primers were designed to amplify an amplicon at a length between 80 and 200 bp. PCR amplification kinetics (efficiency) of each primer were tested prior to experiments with efficiencies between 1.6 and 2. Unspecific binding and dimer formation was minimized by *in silico* analysis prior to experiments. Additionally, for every sample melting curves profiles were analyzed in order to detect unspecific products and primer dimers and all controls consisting of ddH_2_O were negative for target and housekeeping genes as expected. The efficiency-corrected quantitation was performed automatically by the LightCycler 480 based on extern standard curves describing the PCR efficiencies of the target and the reference genes (ratio = *E*_target_*ΔCP*_target_ (control − sample)/*E*_ref_*ΔCP*_ref_ (control − sample)). The high confidence algorithm was applied in order to minimize the risk of false-positive crossing point (*Cp*). If samples did not rise above the background fluorescence (*Cp* or quantitation cycle *Cq*) of 35 cycles during the amplification reaction they were regarded as not detectable, whereas samples with *Cp* between 5 and 35 cycles were regarded as detectable [57].

### Histopathology

After killing the mice, one middle part of each kidney was directly transferred into 4% neutral-buffered formalin for fixation. After 12 h, the tissues were processed by dehydrating in graded alcohol and by embedding in paraffin for long-term storage. From paraffin blocks, 4-μm sections were generated and further processed by deparaffinizing, rehydrating, and transferring into citrate buffer. Microwave treatment or autoclaving was performed for antigen retrieval by established protocols [58]. For immunostaining, the following primary antibodies were used: anti-IL-22 (Santa Cruz Biotechnology), anti-THP (Cedarline), anti-neutrophil/Ly6B.2 (Serotec), anti-F4/80 (Serotec). Histopathologic stainings were assessed by a blinded observer and trends (more, unchanged, less staining/positive cells) were defined arbitrarily based on differential staining intensity/cell counts compared with healthy tissues.

### Statistics

Data are shown as mean ± S.E.M. Univariate ANOVA (a value of *P*<0.05 indicated statistical significance) was used to assess comparison between groups and post hoc Bonferroni’s correction was applied in order to correct multiple comparisons. In order to assess correlation between murine and mouse tissue samples, Pearson’s r was calculated using GraphPad Prism.

## Supporting information

**Suppl. Figure 1.** Log2- fold change of gene expression for rIRI. Bar graphs showing expression values of Notch/Ahr/IL-22 axis genes 24 hrs following different ischemia times (“dosage”, Figure 1a) and gene expression at different timepoints after 35 min of ischemia (“timeline”, Figure1b). bar graphs show means and SEM, respectively.

**suppl. Table 1:** Cohort of humans

**suppl. Table 2:** Values of different housekeeping gene for human organ panel

**suppl. Table 3:** Values of housekeeping gene 18s

**suppl. Table 4:** Murine Primers

**suppl. Table 5:** Human Primers

## Abbreviations

AhR: aryl hydrocarbon receptor
*Cp*: crossing point
IL-22: interleukin-22
IRI: ischemia–reperfusion injury
PAS: periodic acid–Schiff’s staining
rAOC: acute renal oxalate crystallopathy
rCOC: renal chronic oxalate crystallopathy
rIRI: renal IRI
THP: Tamm–Horsfall protein
UUO: unilateral ureteral obstruction
RT-qPCR: reverse transcription quantitative PCR
PCR: polymerase chain reaction
