# Supplementary material for: Gene expression profiling of the Notch-AhR-IL22 axis at homeostasis and in response to tissue injury
Source: Biosci Rep. 2017 Dec 22;37(6):BSR20170099. doi: 10.1042/BSR20170099 (PMC5741834; doi:10.1042/BSR20170099)
Supplement: Supplementary file 1 [file bsr20170099_Supp1.pdf]

suppl. Table 1 Cohort of humans

Tissue	Source	Number of individuals
Heart	pooled from 3 male Causcasians, ages: 30, 30, 39	3
Bone marrow	pooled from 4 male/female Caucasians, ages: 58 - 76	4
Kidney	40-year old female caucasian	1
Liver	pooled from 3 male Asians, ages: 24 - 64	3
Lung	pooled from 3 male/female Caucasians, ages: 32-61	3
Spleen	pooled from 15 male/female Caucasians, ages: 22-69	15
Testis	pooled from 7 Asian/Caucasions:24 - 87	7
Thymus	pooled from 2 Caucasians: ages: 18-57	2
Colon	pooled from 3 male Asians, ages: 24 - 29	3
Brain	pooled from 4 male Asians, ages: 21- 29	4

suppl. Table 2 Values of different housekeeping genes for human organ panel

	GAPDH	B-Actin	HPRT	18s
N	10.00	10.00	10.00	10.00
GM	20.11	16.40	24.14	7.75
AM	20.15	16.42	24.19	7.77
Min	18.62	15.52	21.05	6.75
Max	22.07	18.60	26.13	8.54
SD	1.27	0.91	1.55	0.63
Variance	1.62	0.82	2.41	0.40
CV	0.06	0.06	0.06	0.08

suppl. Table 3 Values of housekeeping gene 18s

**18s quality control**	**human tissues**	**murine tissues**	**IRI**	**CaOx**	**UUO**
**NO of samples**	10.00	20.00	22.00	20.00	12.00
**GM**	7.75	10.07	8.75	10.08	10.80
**AM**	7.77	10.22	8.78	10.09	10.82
**Min**	6.75	8.73	7.48	9.49	10.06
**Max**	8.54	15.89	9.98	11.00	12.09
**SD**	0.63	1.93	0.74	0.46	0.62
**Variance**	0.40	3.73	0.55	0.21	0.39
**CV**	0.08	0.19	0.08	0.05	0.06

Suppl. Table 4 Murine Primers

Murine	Forward (5'-3')	Reverse (5'-3')
18s	GCAATTATTCCCCATGAACG	AGGGCCTCACTAAACCATCC
DLL1	GCTGGAAGTAGATGAGTGTGCTC	CACAGACCTTGCCATAGAAGCC
DLL3	CCAGCACTGGATGCCTTTTACC	ACCTCACATCGAAGCCCGTAGA
DLL4	GGGTCCAGTTATGCCTGCGAAT	TTCGGCTTGGACCTCTGTTCAG
DLK1	TACCCTAGCCCAAGACTCCA	TACCCTAGCCCAAGACTCCA
DLK2	GGAGGCCACTGTGTGTATGA	CGTCCACATTGACCTCACAG
JAG1	TGCGTGGTCAATGGAGACTCCT	TCGCACCGATACCAGTTGTCTC
JAG2	CGCTGCTATGACCTGGTCAATG	TGTAGGCGTCACACTGGAACTC
NOTCH1	GCTGCCTCTTTGATGGCTTCGA	CACATTCGGCACTGTTACAGCC
NOTCH2	CCACCTGCAATGACTTCATCGG	TCGATGCAGGTGCCTCCATTCT
NOTCH3	GGTAGTCACTGTGAACACGAGG	CAACTGTCACCAGCATAGCCAG
NOTCH4	GGAGATGTGGATGAGTGTCTGG	TGGCTCTGACAGAGGTCCATCT
ADAM17	TGTGAGCGGTGACCACGAGAAT	TTCATCCACCCTGGAGTTGCCA
PSEN1	GAGACTGGAACACAACCATAGCC	AGAACACGAGCCCGAAGGTGAT
BSG	ACATAGTGGACGCAGATGACC	GCGTGGATACCACCACATACT
RBP-J	TGGCTACATCCATTACGGGCAG	GTGGAGTTGTGATACAGGGTCG
HES1	GGAAATGACTGTGAAGCACCTCC	GAAGCGGGTCACCTCGTTCATG
HES5	GTGACTTCTGCGAAGTTCCTG	GGCCCTGAAGAAAGTCCTCTA
HEY1	CCAACGACATCGTCCCAGGTTT	CTGCTTCTCAAAGGCACTGGGT
HEYL	CTGGAGAAAGCTGAGGTCTTGC	ACCTCAGTGAGGCATTCCCGAA
AHR	CTGGTTGTCACAGCAGATGCCT	CGGTCTTCTGTATGGATGAGCTC
ARNT	CTCACGAAGGTCGTTCATCTGC	CCACAAAGTGAGGTTCTCCTTCC
ARNT2	GAAGACGCTGATGTCGGACAAG	CAGAGTTGTGCCGTGACAGGAA
CYP1A1	GACCCTTACAAGTATTTGGTCGT	GGTATCCAGAGCCAGTAACCT
CYP24A1	CTGCCCCATTGACAAAAGGC	CTCACCGTCGGTCATCAGC
IL22	TGGGATTTGTGTGCAAAAGCA	TAATTTCCAGTCCTGTCTTCTG
IL22RA1	CTACGTGTGCCGAGTGAAGA	AAGCGTAGGGGTTGAAAGGT
IL22RA2	CCAAACCAGTCTGAGAGCACCT	CAGGACAATGCCTGAGCCTTTC
IL10R2	TGCTTCTCCGTCCTCCAGAGTT	GCTCTCTGAGTTCCTTCATAGGC
STAT3	AGGAGTCTAACAACGGCAGCCT	GTGGTACACCTCAGTCTCGAAG

Suppl. Table 5 Human Primers

Human	Forward (5'-3')	Reverse (5'-3')
18s	GCAATTATTCCCCATGAACG	AGGGCCTCACTAAACCATCC
DLL1	TGCCTGGATGTGATGAGCAGCA	ACAGCCTGGATAGCGGATACAC
DLL3	CACTCAACAACCTAAGGACGCAG	GAGCGTAGATGGAAGGAGCAGA
DLL4	CTGCGAGAAGAAAGTGGACAGG	ACAGTCGCTGACGTGGAGTTCA
DLK1	AAG GAC TGC CAG AAA AAG GAC	GCA GAA ATT GCC TGA GAA GC
DLK2	GGCAGGCAAGTTCTGTGACAAAG	CATGGAAGCCTGGTAAGCACAC
JAG1	TGCTACAACCGTGCCAGTGACT	TCAGGTGTGTCGTTGGAAGCCA
JAG2	GCTGCTACGACCTGGTCAATGA	AGGTGTAGGCATCGCACTGGAA
NOTCH1	GGTGAACTGCTCTGAGGAGATC	GGATTGCAGTCGTCCACGTTGA
NOTCH2	GTGCCTATGTCCATCTGGATGG	AGACACCTGAGTGCTGGCACAA
NOTCH3	TACTGGTAGCCACTGTGAGCAG	CAGTTATCACCATTGTAGCCAGG
NOTCH4	TTCCACTGTCCTCCTGCCAGAA	TGGCACAGGCTGCCTTGGAATC
ADAM17	AACAGCGACTGCACGTTGAAGG	CTGTGCAGTAGGACACGCCTTT
PSEN1	GCAGTATCCTCGCTGGTGAAGA	CAGGCTATGGTTGTGTTCCAGTC
BSG	GGCTGTGAAGTCGTCAGAACAC	ACCTGCTCTCGGAGCCGTTCA
RBP-J	TCATGCCAGTTCACAGCAGTGG	TGGATGTAGCCATCTCGGACTG
HES1	GGAAATGACAGTGAAGCACCTCC	GAAGCGGGTCACCTCGTTCATG
HES5	TTGTTCTGTGTTTGCATTTAAG	AGAAAGTCCTCTACAGGCTG
HEY1	TGTCTGAGCTGAGAAGGCTGGT	TTCAGGTGATCCACGGTCATCTG
HEYL	TGGAGAAAGCCGAGGTCTTGCA	ACCTGATGACCTCAGTGAGGCA
AHR	GTCGTCTAAGGTGTCTGCTGGA	CGCAAACAAAGCCAACTGAGGTG
ARNT	CTGTCATCCTGAAGACCAGCAG	CTGGTTCTCATCCAGAGCCATTC
ARNT2	GGAATGCCTACTCCAGTCTTGC	CTTTGCCACTGCGACCAGACTT
CYP1A1	GATTGAGCACTGTCAGGAGAAGC	ATGAGGCTCCAGGAGATAGCAG
CYP24A1	CATCATGGCCATCAAAACAAT	GCAGCTCGACTGGAGTGAC
IL22	TCACCCTTGAAGAAGTGCTGT	ACATGTGCTTAGCCTGTTGCT
IL22RA1	GGTCAACCGCACCTACCAAATG	TGATGGTGCCAAGGAACTCTGTG
IL22RA2	GCCGAAAGAAGCTACCCAGTGT	GGTCCAAGTTCTTCAGCTCTGG
IL10R2	GGCTTCCTCATCAGTTTCTTCC	TTCCACACATCTCTCTTCACTTCTC
STAT3	CTTTGAGACCGAGGTGTATCACC	GGTCAGCATGTTGTACCACAGG

Suppl. Figure 1. Log2- fold change of gene expression for rIRI. Bar graphs showing expression values of Notch/Ahr/IL-22 axis genes 24 hrs following different ischemia times ("dosage", Figure 1a) and gene expression at different timepoints after 35 min of ischemia ("timeline", Figure1b). bar graphs show means and SEM, respectively.
